# A Case of Late Diagnosis of Latent Autoimmune Diabetes in Adults

**DOI:** 10.7759/cureus.21826

**Published:** 2022-02-02

**Authors:** Joana R Costa, Ana Mestre, Mariana S Miranda, Frederica H Ferreira, Yahia Abuowda

**Affiliations:** 1 Family Medicine, Unidade de Saúde Familiar Vale de Sorraia, Santarém, PRT; 2 Internal Medicine, Hospital Distrital de Santarém, Santarém, PRT

**Keywords:** c-peptide, insulin, latent autoimmune diabetes, autoantibodies, diabetes mellitus

## Abstract

Latent autoimmune diabetes of adults (LADA) is a type of autoimmune diabetes that begins in adulthood (usually after the age of 35 years); its main feature is the presence of diabetes-associated autoantibodies (most often autoantibody against glutamic acid decarboxylase), which leads to progressive destruction of the islets of Langerhans. This is a heterogeneous condition that presents with clinical and laboratory manifestations common to type 1 diabetes and type 2 diabetes.

We report a case of a 71-year-old man diagnosed with type 2 diabetes two years ago, poorly controlled with oral antidiabetic therapy, and worsening in the third year. He had a positive family history of type 2 diabetes in two second-degree relatives (nephews). No pathologic findings at the physical examination were found. His body mass index was 23 kg/m^2^ and glycated hemoglobin was 10.6%. Laboratory workup revealed low basal C-peptide (<0.1 ng/mL) and positive glutamic acid decarboxylase antibodies, and the LADA diagnosis was confirmed.

This case highlights the importance of being aware of this disease, especially in patients previously diagnosed with type 2 diabetes who remain uncontrolled with diet and oral hypoglycemic agents. LADA is often confused with type 2 diabetes, and therefore, the management is frequently inadequate. An early diagnosis and treatment are crucial to delaying disease progression.

## Introduction

Diabetes mellitus (DM) remains one of the commonest chronic diseases worldwide, and it is among the top 10 causes of death in adults [1]. Although the classification of DM has been expanding, traditionally, a newly diagnosed patient receives a diagnosis of either type 1 or 2 diabetes based on the clinical scenario. Type 1 DM results from an autoimmune process leading to the destruction of β-cells in the pancreas, and consequentially, insulin is absent or extremely low. In contrast, type 2 diabetes is characterized by elevated insulin levels and insulin resistance, commonly developed from obesity and aging [2]. However, a small fraction of the patients diagnosed with diabetes in adult life present features of these two types of DM.

Latent autoimmune diabetes of adults (LADA) accounts for 2-12% of all cases of diabetes in the adult population (with variability according to ethnicity and method used for screening), and it is characterized by a slow and progressive immune-mediated destruction of the islets of Langerhans [3]. The initial course of LADA clinically resembles type 2 diabetes due to the age of onset and typically does not require insulin therapy in the short term after diagnosis. However, LADA also presents autoimmune characteristics like type 1 diabetes, with the presence of autoantibodies against pancreatic islet-cells such as the autoantibody against glutamic acid decarboxylase (GADA) [4]. 

The Immunology of Diabetes Society (IDS) has established three main criteria, including (1) adult age of onset (>30 years), (2) presence of at least one islet-cell autoantibody, and (3) absence of insulin requirement for at least six months after diagnosis, to diagnose LADA [5]. Nonetheless, the definition of LADA remains controversial because this entity is clinically and metabolically a hybrid of type 1 and type 2 diabetes, and it is challenging to define categorical immunogenetic and phenotypic features [6]. Herein, we report a case of a 71-year-old male previously diagnosed with type 2 diabetes but whose characteristics and progression led to the correct diagnosis of LADA.

## Case presentation

A 71-year-old Caucasian male was diagnosed with type 2 diabetes one year ago, and he was started on metformin, with the later addition of vildagliptin and acarbose. Despite dietary measures and oral antidiabetic therapy, the patient remained poorly controlled, and he was referred to an internal medicine diabetic clinic. In addition to diabetes, the patient had essential hypertension and was medicated with telmisartan.

At the initial visit to diabetic clinic, the patient reported no improvement from current oral medications. The fasting blood sugar ranged from 110 to 300 mg/dL and postprandial from 200 to 310 mg/dL, and his glycated hemoglobin (A1C) was 10.6%. His body weight was 67 kg, and his body mass index (BMI) was 23 kg/m^2^. His arterial blood pressure was 121/73 mmHg. On physical examination, no signs of adenopathies, hepatosplenomegaly, thyroid pathology, or other pathologic findings were found. He had a positive family history of type 2 diabetes in two second-degree relatives (nephews), and he had no known family history of autoimmune disease.

Due to a poor response to previous medications, LADA was considered in the differential diagnosis, and the patient was instructed to discontinue oral agents and was started on insulin glargine 18 units daily. Further evaluation evidenced low basal C-peptide levels (<0.1 ng/mL), and the autoantibody against glutamic acid decarboxylase (GADA) was positive (113.9 U/mL). The rest of the laboratory tests showed no microalbuminuria, normal renal and hepatic function, normal electrolytes, and an adequate lipid profile (Table 1). Screening for diabetic retinopathy was negative and abdominal ultrasound revealed no changes.

**Table 1:** Laboratory findings of the patient Hb: hemoglobin; WBC: white blood cell count; PLT: platelets; HDL: high-density lipoproteins; LDL: low-density lipoproteins; GADA: glutamic acid decarboxylase antibody; IAA: Insulin autoantibodies; ICA: islet cell antibody

Laboratory findings	Case report	Reference range
Hb (g/dL)	14.8	13.0-17.0
WBC (10^9^/L)	4.9	4.0-10.0
PLT (10^9^/L)	182	150-400
Urea (mg/dL)	39	18-55
Creatinine (mg/dL)	1.0	0.7-1.3
Serum sodium (mmol/L)	137	136-145
Serum potassium (mmol/L)	4.1	3.4-5.1
Total cholesterol (mg/dL)	182	< 200
LDL (mg/dL)	114	< 130
HDL (mg/dL)	51	> 55
Triglycerides (mg/dL)	82	< 150
Fasting glucose (mg/dL)	255	83-110
Glycated hemoglobin (%)	10.6	4.5-6.5
C-peptide (ng/mL)	< 0.1	0.78-5.19
GADA (U/mL)	113.8	< 5.0
IAA	0.0	< 0.4
ICA	Negative	Negative/Positive

LADA diagnosis was confirmed with the presence of GADA. A therapeutic regimen with glargine basal insulin once daily and insulin lispro before meals was initiated, and the patient was educated about counting carbohydrates to further match insulin doses to carbohydrate intake. At follow-up three and nine months later, the patient maintained reasonable glycemic control, and his hemoglobin A1C had improved to 8.6% and 6.3%, respectively. The patient confirmed adherence to his insulin regimen and carbohydrate counting at meals, and his glucose remained controlled, continuing the same therapy.

## Discussion

LADA, also called type 1.5 diabetes, is the most common form of autoimmune diabetes diagnosed in adults, and approximately 5-12% of cases in European populations with apparent type 2 diabetes are, in fact, misdiagnosed LADA patients [7]. This disease is part of a continuum between type 1 and type 2 diabetes. LADA has a genetic and immunologic profile similar to type 1 diabetes but clinical manifestations resembling type 2 diabetes. In type 1 DM, an autoimmune response directed against pancreatic islet cells leads to β-cell destruction, resulting in insulin insufficiency. However, LADA patients do not immediately lose all β-cell function, and maybe this is why most of these patients have a clinical presentation that looks like type 2 DM [8]. At the time of diagnosis, insulin secretion is often similar in patients with LADA and type 2 diabetes. Initially, LADA patients may have enough β-cell function to be controlled only with diet and oral antidiabetic therapy; however, over time, they develop insulin insufficiency and thus often require insulin [9]. Compared to type 2 DM, LADA patients require insulin treatment earlier and more commonly short-term after diagnosis [9,10].

A critical feature to identify LADA is the presence of islet autoantibodies, the same used to identify type 1 diabetes. GADA is the most sensitive marker in LADA, whereas islet-cell antibodies (ICA), protein tyrosine phosphatase autoantibodies (IA-2A), insulin autoantibodies (IAA), and islet-specific zinc transporter isoform 8 (ZnT8) autoantibodies are positive only in a small fraction of these patients [11]. In the action LADA study, approximately 90% of LADA subjects with diabetes-associated autoantibodies are GADA positive [3].

The measurement of C-peptide, which is used as a marker of β-cell function, can be helpful in clinical practice. In type 1 diabetes, C-peptide is frequently undetectable (suggesting insulin deficiency due to β-cell destruction); in contrast, type 2 diabetes usually presents with normal or elevated levels (reflecting hyperinsulinemia due to compensatory increase in insulin secretion caused by insulin resistance); and in LADA patients C-peptide levels are low or normal [12]. C-peptide can be used to stage LADA patients according to their residual β-cell function and, therefore, their progression toward insulin requirement [6].

Another essential feature in diabetic patients is the concomitant presence of metabolic syndrome. LADA patients showed a higher prevalence of metabolic syndrome components than type 1 diabetic subjects [13]. In contrast, they generally have a better metabolic profile than those with type 2 diabetes, with lower triglyceride, higher HDL cholesterol levels, and lower blood pressure and BMI [9,12,14]. In fact, one study showed that BMI values of LADA patients were significantly lower than patients with type 2 diabetes, confirming the nonobese nature of these patients [12].

In the presented case, the patient fits the proposed IDS criteria for diagnosing LADA - age of onset >30 years, the presence of islet-cell autoantibody (in this case, GADA was markedly positive), and he was not treated with insulin within the first six months after diagnosis. Additionally, the patient had a low C-peptide level and a BMI <25 kg/m^2^. Furthermore, the progression of the disease raised a high suspicion of being a LADA patient, he was diagnosed initially with type 2 diabetes treated accordingly with oral antidiabetics (and he did not require insulin for at least six months after diagnosis); however, despite adherence, he rapidly required insulin therapy.

In most of the case reports on LADA, patients usually present under the age of 60 years, and some authors consider age under 50 years as a clinical feature of LADA [15]. However, there is no precise age that defines a typical age of onset. This case highlights that LADA is characterized by significant degree of phenotypic heterogeneity, and despite age, patients with a high index of suspicion should get tested for autoantibodies. Table 2 presents the main features of LADA, type 1 and type 2 diabetes, and the characteristics of this case report.

**Table 2:** Differences between LADA, type 1 and type 2 diabetes, and the present case report. LADA: latent autoimmune diabetes of adults; DM: diabetes mellitus

Characteristics	Type 1 DM	Type 2 DM	LADA	Case report
Age of onset (years)	Usually childhood/adolescence	Adulthood (rarely before)	>30	71
Pancreatic autoantibodies	Positive	Negative	Positive	Positive
C -peptide levels at diagnosis	Absent or low	Normal to increased	Low to normal	Low
Insulin requirement	At diagnosis	Absent or years after diagnosis	>6 months after diagnosis	12 months after onset
Metabolic syndrome	Uncommon	Frequently present	Uncommon	Not present
Family history of diabetes	Negative or positive	Frequently positive	Negative or positive	Nephews with type 2 DM
Personal or family history of autoimmunity	Frequently present	Absent	Frequentlty present	Absent

LADA is a well-recognized form of autoimmune diabetes; however, there are no established guidelines for its management. The overall objective of a correct therapeutic strategy for LADA patients should aim to the preservation of residual β-cell function as well as to achieve reasonable metabolic control to reduce the risk of long-term complications [4].

Insulin therapy is effective and safe for LADA patients, as it preserves pancreatic β-cell function. However, it remains uncertain if insulin should be administered in the early stages of the disease, especially when substantial β-cell function is present. Therefore, in 2020, Buzzetti et al. proposed a personalized approach for LADA management, driven by the measurement of C-peptide [6]. The group recommended measuring C-peptide levels, which are directly proportional to levels of insulin production, for evaluating β-cell function. Then, if C-peptide levels are <0.3 nmol/L, a multiple-insulin regimen is recommended, and if C-peptide levels >0.3 and <0.7 nmol/L, insulin in combination with other therapies to control/ prevent diabetic complications should be considered. Finally, if C-peptide levels >0.7 nmol/L, the group suggests using a slightly modified American Diabetes Association/European Association for the Study of Diabetes (ADA/EASD) algorithm for type 2 DM by monitoring C-peptide to adjust treatment if necessary.

In addition to insulin, other therapy options that preserve β-cell function, including dipeptidyl peptidase 4 inhibitors and glucagon-like peptide 1 receptor agonists, could be considered for LADA patients [16]. In a small study, thiazolidinediones, when combined with insulin, preserved β-cell function in LADA; however, the potential side effects (risk of atypical bone fractures, macular edema, and weight gain) could be a limitation for the use of these oral agents [6,17]. Sulfonylureas should not be used as first-line therapy in patients with LADA; these oral hypoglycemic agents were associated with worse metabolic control and more rapid deterioration of C-peptide secretion compared with insulin [18].

Like in type 2 diabetes, lifestyle modifications, including diet and exercise programs targeted to individual needs, might play an important role in LADA management. However, additional studies are needed to clarify the influence of lifestyle factors on the risk of LADA [19].

## Conclusions

This case highlights the importance of the clinical recognition of LADA and the implementation of screening diagnostic tests, emphasizing that all patients who do not fit the typical type 2 DM profile should be further investigated. The early identification of these patients is associated with better glycemic control, thus potentially decreasing the risk of long-term complications. The best therapeutic approach should be individualized and tailored to each patient, considering the importance of C-peptide measurement to begin insulin therapy.
